# Prevalence of Steatotic Liver Disease Based on a New Nomenclature in the Japanese Population: A Health Checkup-Based Cross-Sectional Study

**DOI:** 10.3390/jcm13041158

**Published:** 2024-02-19

**Authors:** Takao Miwa, Satoko Tajirika, Nanako Imamura, Miho Adachi, Ryo Horita, Tatsunori Hanai, Taku Fukao, Masahito Shimizu, Mayumi Yamamoto

**Affiliations:** 1 Health Administration Center, Gifu University, Gifu 501-1193, Japan; 2 Department of Gastroenterology/Internal Medicine, Graduate School of Medicine, Gifu University, Gifu 501-1194, Japan; 3 United Graduate School of Drug Discovery and Medical Information Sciences, Gifu University, Gifu 501-1194, Japan

**Keywords:** lean, metabolic dysfunction-associated fatty liver disease, nonalcoholic fatty liver disease, nonalcoholic steatohepatitis, nonobese, obesity

## Abstract

This cross-sectional study examined the prevalence and characteristics of steatotic liver disease (SLD) based on a recently introduced nomenclature in the Japanese health checkup population. SLD was evaluated using liver ultrasonography, and participants were categorized into metabolic dysfunction-associated steatotic liver disease (MASLD), metabolic dysfunction and alcohol associated steatotic liver disease (MetALD), alcohol-associated/related liver disease (ALD), and cryptogenic SLD groups. The prevalence and characteristics of the SLD subclasses were assessed, and subgroup analyses were conducted for the non-obese (body mass index [BMI] ≤ 25 kg/m^2^) and lean (BMI ≤ 23 kg/m^2^) populations. Among the 694 participants, with a median age of 47 years and comprising 54% males, the prevalence of MASLD, MetALD, ALD, and cryptogenic SLD was 26%, 2%, 1%, and 2%, respectively. A remarkable difference was observed in the prevalence of SLD subclasses according to age, sex, and BMI. Subgroup analyses revealed heterogeneous demographic, clinical, and biochemical parameters between the SLD categories. Individuals with MetALD had higher gamma-glutamyl transferase levels, lower platelet counts, and higher fibrosis-4 index than did those with MASLD. Furthermore, the prevalence of non-obese and lean MASLD was 13% and 6%, respectively. This study provides preliminary information on the prevalence of SLD based on a new nomenclature in the Japanese population.

## 1. Introduction

For many years, obesity, type 2 diabetes, and cardiometabolic risks have been widely accepted as robust factors associated with nonalcoholic fatty liver disease (NAFLD) [1,2]. Although accumulated evidence has shown the importance of NAFLD, the use of stigmatizing terms to explain steatotic liver disease (SLD) has gradually raised the requirement for renaming the term to a broadly acceptable nomenclature. Furthermore, because the pathophysiology of NAFLD and that of alcohol-associated/related liver disease (ALD) share similar biological processes, there is a need to clearly define the contribution of alcohol to the definition of SLD to avoid a mixture of etiologies. With these raised concerns, global academic experts have recently suggested a new nomenclature for SLD, namely, metabolic dysfunction-associated steatotic liver disease (MASLD), metabolic dysfunction and alcohol associated steatotic liver disease (MetALD), ALD, and cryptogenic SLD [3].

The new nomenclature for SLD not only renamed the term but also changed the diagnostic approach for SLD. Indeed, although NAFLD is diagnosed with the exclusion of other etiologies, MASLD is diagnosed with the inclusion of cardiometabolic criteria in those with mild alcohol consumption [3]. Furthermore, there is a clear definition of MetALD, which includes both cardiometabolic risks and moderate alcohol consumption, distinct from ALD, with excessive alcohol consumption [3]. Several studies have assessed the impact of each SLD on outcomes since the suggestion of the new nomenclature; however, data regarding the prevalence of SLD and its subclassification, such as MASLD, MetALD, ALD, and cryptogenic SLD, are limited, especially in the general Japanese population.

Thus, we sought to comparatively investigate the prevalence and characteristics of MASLD, MetALD, ALD, and cryptogenic ALD among the Japanese population using health checkup data.

## 2. Materials and Methods

### 2.1. Study Design and Participants

**Figure 1:**
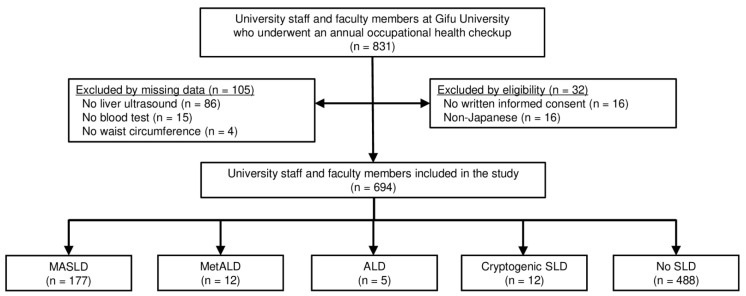
A flow diagram of the study. Abbreviations: ALD, alcohol-associated/related liver disease; MASLD, metabolic dysfunction-associated steatotic liver disease; MetALD, metabolic dysfunction and alcohol associated steatotic liver disease; SLD, steatotic liver disease.

This cross-sectional study evaluated 831 university staff and faculty members who underwent annual occupational health checkups at Gifu University (a general and national university in Gifu, Japan) in June 2023, which was mandatory based on the Occupational Health and Safety Act. The final study included 694 participants based on the inclusion and exclusion criteria (Figure 1). The inclusion criteria were university staff and faculty members who underwent a health checkup and provided written informed consent. The exclusion criteria were self-identification as non-Japanese and missing data. The study protocol was reviewed and approved by the Institutional Review Board of the Graduate School of Medicine, Gifu University (approval no. 2022-237), and conformed to the provisions of the Declaration of Helsinki.

### 2.2. Clinical and Biochemical Evaluations

Clinical and laboratory variables were assessed using health checkup data. Alcohol intake was self-reported through a questionnaire. The participants were asked, “How many days per week do you consume alcohol on average?” with choices ranging from 0 days to every day. Additionally, they were asked, “How many cups of sake do you consume at one drinking session?” with options from less than 1 to more than 3 cups. A cup (180 mL) of sake is equivalent to one-quarter of a bottle of wine or 180 mL of 14% alcohol. Daily alcohol intake was calculated based on the frequency and amount of consumption per session. Based on the questionnaire, alcohol intake was categorized as none, mild (≤210 g/week for males and ≤140 g/week for females), moderate (between 210 and 420 g/week for males and 140–350 g/week for females), and excessive (≥420 g/week for males and ≥350 g/week for females) based on the consensus statement [3]. Weight, height, and waist circumference were measured at the time of the health checkup. Body mass index (BMI) was calculated by dividing weight by the square of height, and the participants were categorized as obese (BMI ≥ 25 kg/m^2^) and non-obese (<25 kg/m^2^) based on the reference data suggested by the Japan Society for the Study of Obesity [4], or overweight (≥23 kg/m^2^) and lean (<23 kg/m^2^) based on the cutoff values determined in the new SLD consensus statement [3].

Biochemical parameters included aspartate aminotransferase (AST), alanine aminotransferase (ALT), gamma-glutamyl transferase (GGT), creatinine, triglyceride (TG), high-density lipoprotein (HDL) cholesterol, low-density lipoprotein (LDL) cholesterol, hemoglobin A1c (HbA1c), fasting or occasional glucose levels, and platelet count. To assess liver fibrosis, the fibrosis-4 (FIB-4) index was calculated using the following formula: ((age [years]) × (AST [IU/L]))/((platelet count [10^9^/L]) × (ALT [IU/L])^1/2^) [5].

### 2.3. Assessment of SLD Based on the New Nomenclature

Participants were evaluated for SLD using liver ultrasound (LOGIQ P10; GE Healthcare Japan, Tokyo, Japan) with the following findings: bright liver, hepatorenal echo contrast, hepatosplenic echo contrast, attenuation, and vascular blurring [6,7]. Those with SLD were categorized into MASLD, MetALD, ALD, and cryptogenic SLD based on the new nomenclature for SLD [3]. MASLD was diagnosed when SLD was present along with any of the following cardiometabolic criteria: overweight (BMI ≥ 23 kg/m^2^), elevated waist circumference (waist circumference >94 cm in males and >80 cm in females), impaired glucose tolerance (fasting serum glucose level ≥100 mg/dL, occasional glucose level ≥140 mg/dL, HbA1c ≥ 5.7%, presence of type 2 diabetes), hypertension (blood pressure ≥130/85 mmHg), hypertriglyceridemia (plasma TG ≥ 150 mg/dL), low HDL cholesterol (plasma HDL cholesterol <40 mg/dL), or any self-reported treatment related to these conditions. MetALD was diagnosed when patients with MASLD had moderate alcohol intake. ALD was diagnosed when the participants had SLD and excessive alcohol intake. Those with SLD without any cardiometabolic criteria, excessive alcohol intake, or other causes were categorized as having cryptogenic SLD.

### 2.4. Statistical Analyses

Continuous and categorical variables are expressed as the median (interquartile range) and number of patients (percentage), respectively. The groups were compared using the chi-square test or Kruskal–Wallis test. For continuous variables, multiple pairwise comparisons were performed using the Steel–Dwass test considering the MASLD group as the reference to elucidate the clinical characteristics of those with MASLD compared to those without SLD and to those with other SLD subclassifications. Given the reference cutoff values of obesity and overweight, the prevalence and characteristics of SLD among non-obese and lean subgroups were also reported [3,4]. All analyses were two-sided, and *p* < 0.05 was set as the threshold for statistical significance. All analyses were performed using JMP Pro (version 17.0.0; SAS Institute Inc., Cary, NC, USA).

## 3. Results

### 3.1. Characteristics of Participants

The participants’ characteristics are listed in Table 1. The median age of the 694 participants was 47 years and 54% were males. The prevalence of mild, moderate, and excessive alcohol consumption was 40%, 7%, and 2%, respectively. The median waist circumference and BMI were 78 cm and 23 kg/m^2^, respectively. Regarding the cardiometabolic criteria, the prevalence of overweight, elevated waist circumference, impaired glucose tolerance, hypertension, hypertriglyceridemia, and low HDL cholesterol was 48%, 45%, 20%, 29%, 32%, and 17%, respectively. 

### 3.2. Prevalence and Characteristics of SLD in the Total Population

**Figure 2:**
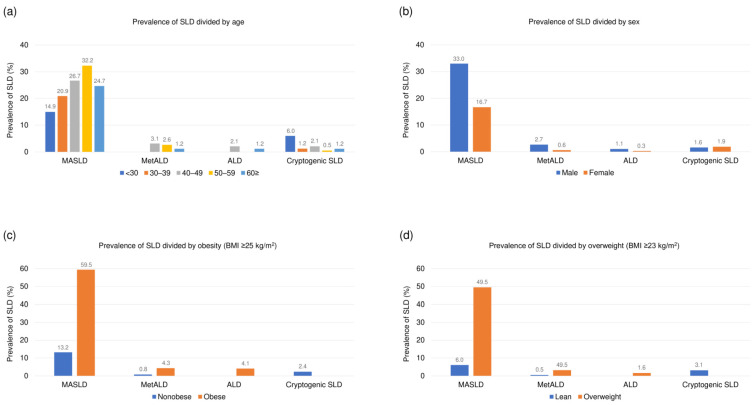
Prevalence of SLD based on the new nomenclature divided by (**a**) age, (**b**) sex, (**c**) obesity, and (**d**) overweight. Abbreviations: ALD, alcohol-associated/related liver disease; BMI, body mass index; MASLD, metabolic dysfunction-associated steatotic liver disease; MetALD, metabolic dysfunction and alcohol associated steatotic liver disease; SLD, steatotic liver disease.

A comparison of the SLD subclasses is shown in Table 1. The prevalence of MASLD, MetALD, ALD, and cryptogenic SLD was 26% (n = 177), 2% (n = 12), 1% (n = 5), and 2% (n = 12), respectively. The bar graphs show a robust relationship between age (Figure 2a), sex (Figure 2b), obesity (Figure 2c), overweight (Figure 2d), and the prevalence of SLD. Comparisons between the SLD subclasses showed significant differences in demographic, clinical, and biochemical parameters (all *p* < 0.05). The biochemical parameters were generally worse in patients with MASLD than in those with cryptogenic SLD or without SLD. Notably, multiple pairwise comparisons revealed that participants with MetALD had significantly higher GGT levels, lower platelet counts, and higher FIB-4 index values than did those with MASLD (Table 1).

### 3.3. Prevalence and Characteristics of SLD in the Non-Obese (<25 kg/m^2^) Population

The prevalence of SLD and comparisons between groups among non-obese participants are shown in Table 2. Among 509 non-obese participants, the prevalence of MASLD, MetALD, and cryptogenic SLD was 13% (n = 67), 1% (n = 4), and 2% (n = 12), respectively. No ALD was observed in non-obese participants. The comparisons between all groups showed significant differences in demographic, clinical, and biochemical parameters, except for age and FIB-4 index (all *p* < 0.05). Notably, multiple pairwise comparisons showed that all biochemical parameters were worse in the participants with MASLD than in those without SLD (Table 2).

### 3.4. Prevalence and Characteristics of SLD in the Lean (<23 kg/m^2^) Population

The prevalence of SLD and comparisons between groups among lean participants are shown in Table 3. Among 383 lean participants, the prevalence of MASLD, MetALD, and cryptogenic SLD was 6% (n = 23), 1% (n = 2), and 3% (n = 12), respectively. No ALD was observed among the lean participants. The comparisons between all groups showed that serum ALT, GGT, TG, HDL cholesterol, LDL cholesterol, and HbA1c levels significantly differed between the groups (all *p* < 0.05). Notably, multiple pairwise comparisons showed that serum TG and HbA1c levels were higher in participants with MASLD than in those with cryptogenic SLD or without SLD (Table 3).

## 4. Discussion

To the best of our knowledge, this study represents the first investigation of the prevalence of SLD according to the new nomenclature in a Japanese health checkup population. The primary finding of this study was the prevalence and clinical characteristics of the SLD subclasses in a Japanese population. Furthermore, we investigated the prevalence and characteristics of SLD in non-obese and lean individuals. The findings of our study highlight the real-world prevalence of SLD in the Japanese population and can guide future studies on SLD in this population. 

With the recent renaming of NAFLD as MASLD, there is a lack of evidence regarding the prevalence and characteristics of SLD in the Japanese population. Although previous studies have mainly focused on the difference between NAFLD and ALD, MetALD was defined as a separate category in the new nomenclature to clarify the effect of moderate alcohol consumption among individuals with MASLD [3]. In our study, the prevalence of MASLD, MetALD, ALD, and cryptogenic SLD was 26%, 2%, 1%, and 2%, respectively. The results showed that SLD in the Japanese population was mostly MASLD, and the involvement of alcohol, namely, MetALD and ALD, was observed in very few participants. Our results are consistent with those of previous studies. In studies from the U.S. using the National Health and Nutrition Examination Survey data, which was intended to collect general population data, the prevalence of MASLD, MetALD, ALD, and cryptogenic SLD was around 31.3–38.7%, 2.7–2.8%, 0.07–0.2%, and 0.01–0.03%, respectively [8,9]. Furthermore, recent studies investigating SLD in a general Korean population showed that the prevalence of MASLD, MetALD, and ALD was around 27.5–47.2%, 4.4–6.4%, and 1.5–2.1%, respectively [10,11]. Although the Korean studies included a large number of participants, these studies used the fatty liver index but not liver ultrasound to assess SLD [10,11]. In the Japanese population, a recent study revealed that the prevalence of MASLD is 5.9% in older patients with hepatitis C after sustained virologic response [12]. In addition, another Japanese study including Japanese patients with NAFLD showed that 93% of those with NAFLD met the criteria of MASLD [13]. Since previous studies have largely focused on MASLD and excluded those with alcohol consumption, our study without strict exclusion criteria provides novel real-world data on the prevalence of SLD and its subclasses based on the new nomenclature in the general Japanese population. 

Regarding liver fibrosis, the FIB-4 index was significantly lower in individuals with MetALD than in those with MASLD in our study. There is an ongoing discussion on the differences in liver fibrosis between MASLD and MetALD. A study from the U.S. suggested that the effects of MASLD and MetALD on liver fibrosis are similar by comparing the prevalence of advanced fibrosis (≥11.7 kPa) between MASLD and MetALD (7.6 vs. 7.7%) via liver stiffness measurement using vibration-controlled transient elastography [8]. In contrast, another study from the U.S. showed a trend of higher prevalence of an FIB-4 index of ≥2.67 in individuals with MetALD compared to those with MASLD (6.3 vs. 1.5%) [9]. Since accumulated evidence in NAFLD suggests that even an alcohol intake of <210 g/week is associated with advanced fibrosis (odds ratio, 1.56; 95% confidence interval, 1.08–2.26) [14], future studies should investigate the difference between MASLD and MetALD in terms of liver fibrosis and clinical outcomes. Indeed, patients with MetALD have a higher risk of cardiovascular diseases than do those with MASLD (hazard ratio, 1.07; 95% confidence interval, 1.02–1.14) [10]. 

Of note, the median FIB-4 index was relatively low in our study (median, 0.76), even in those with SLD. Given the age-dependency of the FIB-4 index, the relatively young participants could be a reason for the low FIB-4 index in our study. Furthermore, a recent study evaluating a Japanese health checkup population demonstrated higher FIB-4 index values in those without NAFLD than in those with NAFLD [15]. The potential drawback of using the FIB-4 index in the general population can be explained by the elevated ALT and decreased AST/ALT ratio according to liver damage in SLD [15]. Thus, the ability of the FIB-4 index in evaluating liver fibrosis and outcomes within the general health checkup population, predominantly comprising healthy individuals, requires further investigations.

Another important finding of our study was that we sought to explore the prevalence of non-obese and lean MASLD. In our study, the prevalence of non-obese and lean MASLD was 13% and 6%, respectively. Considering a previous meta-analysis that reported the prevalence of non-obese and lean NAFLD as 15.7% and 10.2% [16], the prevalence of non-obese/lean MASLD in our study was slightly lower than that of non-obese/lean NAFLD. This seems reasonable because a previous study revealed that 83.5% of patients with lean NAFLD fulfilled the MASLD criteria [13]. However, further studies are required to investigate the prevalence and characteristics of non-obese/lean MetALD, ALD, and cryptogenic SLD, because the number of participants in these subclasses was small in our study.

However, some limitations of this study must be addressed. First, this single-center study was conducted among Japanese university staff and faculty members, most of whom were desk workers. Therefore, our results may not apply to different ethnicities, professional fields, or age groups. Although the differences in body composition and food culture between regions in Japan are considered to be small, a nationwide survey is necessary to confirm the results of this study. Second, hepatic steatosis by ultrasound and fibrosis by the FIB-4 index were assessed in our study; however, liver biopsy or other imaging techniques to evaluate steatosis and fibrosis were not performed [17]. Third, self-reporting data of alcohol intake and medication use could have underestimated the amount of alcohol consumption and the prevalence of cardiometabolic risks among the participants. Finally, the small number of MetALD, ALD, and cryptogenic ALD cases limited the statistical power of this study. Further studies with larger sample sizes are required to confirm our results. Despite these limitations, the strengths of our study should be emphasized in the assessment of steatosis using liver ultrasound, a generalized method to identify SLD, and the evaluation of MetALD, ALD, and cryptogenic SLD based on the new nomenclature.

## 5. Conclusions

In conclusion, our study provided preliminary information on the prevalence of SLD and its subclasses based on a new consensus nomenclature in the Japanese population.

## Figures and Tables

**Table 1:** Characteristics of participants divided according to the new SLD nomenclature.

Characteristics	Total Participants	MASLD	MetALD	ALD	Cryptogenic SLD	No SLD	*p*-Value
	(n = 694)	(n = 177)	(n = 12)	(n = 5)	(n = 12)	(n = 488)	
Demographics							
Age (years)	47 (36–54)	49 (40–54)	50 (44–57)	48 (46–56)	41 (28–47)	45 (35–54)	0.016
Male sex	376 (54)	124 (70)	10 (83)	4 (80)	6 (50)	232 (48)	<0.001
Alcohol intake, n (%)							<0.001
Mild	275 (40)	82 (46)	0 (0)	0 (0)	4 (33)	193 (39)	
Moderate	52 (7)	0 (0)	12 (100)	0 (0)	1 (8)	40 (8)	
Excessive	13 (2)	0 (0)	0 (0)	5 (100)	0 (0)	8 (2)	
Physical examination							
Waist circumference (cm)	78 (71–85)	88 (82–96)	90 (82–94)	98 (86–111)	75 (67–80) *	75 (68–81) *	<0.001
BMI (kg/m^2^)	23 (20–25)	26 (24–30)	26 (24–27)	26 (24–27)	21 (19–22) *	21 (19–23) *	<0.001
Cardiometabolic criteria							
Overweight, n (%)	330 (48)	158 (89)	10 (83)	5 (100)	0 (0)	157 (32)	<0.001
Elevated waist circumference, n (%)	311 (45)	154 (87)	10 (83)	5 (100)	0 (0)	142 (29)	<0.001
Impaired glucose tolerance, n (%)	136 (20)	65 (37)	3 (25)	1 (20)	0 (0)	67 (14)	<0.001
Hypertension, n (%)	201 (29)	105 (59)	10 (83)	5 (100)	0 (0)	81 (17)	<0.001
Hypertriglyceridemia, n (%)	222 (32)	111 (63)	10 (83)	3 (60)	0 (0)	98 (20)	<0.001
Low HDL cholesterol, n (%)	118 (17)	67 (38)	3 (25)	1 (20)	0 (0)	47 (10)	<0.001
Laboratory test							
AST (IU/L)	18 (15–22)	21 (20–36)	24 (20–36)	22 (20–46)	17 (15–21)	17 (15–21) *	<0.001
ALT (IU/L)	18 (13–26)	27 (19–42)	32 (21–49)	27 (18–52)	18 (15–25) *	15 (12–21) *	<0.001
GGT (IU/L)	20 (15–33)	32 (22–49)	65 (30–149) *	64 (42–181)	20 (15–37)	17 (14–26) *	<0.001
Creatinine (mg/dL)	0.78 (0.67–0.93)	0.85 (0.73–0.98)	0.94 (0.85–1.02)	0.79 (0.64–1.02)	0.71 (0.61–0.84)	0.75 (0.66–0.91) *	<0.001
Triglycerides (mg/dL)	103 (71–163)	171 (113–241)	221 (148–378)	213 (76–397)	73 (61–86) *	88 (61–127) *	<0.001
HDL cholesterol (mg/dL)	60 (49–71)	47 (43–57)	48 (45–64)	66 (55–87)	65 (52–79) *	64 (56–75) *	<0.001
LDL cholesterol (mg/dL)	113 (96–132)	125 (108–148)	123 (104–170)	121 (83–156)	109 (96–141)	109 (91–127) *	<0.001
HbA1c (%)	5.4 (5.2–5.6)	5.5 (5.3–5.8)	5.4 (5.3–5.5)	5.2 (5.1–5.6)	5.2 (5.2–5.4) *	5.3 (5.2–5.5) *	<0.001
Platelet (×10^3^/μL)	259 (227–297)	274 (244–324)	235 (215–304)	290 (243–305)	248 (223–285)	252 (220–288) *	<0.001
FIB-4 index	0.76 (0.56–1.06)	0.69 (0.53–0.87)	0.92 (0.81–1.17) *	1.09 (0.65–1.24)	0.64 (0.49–0.88)	0.76 (0.56–1.06) *	<0.001

Values are presented as numbers (percentages) or medians (interquartile range). * *p* < 0.05, compared to those with MASLD using the Steel–Dwass test for pairwise comparisons. Abbreviations: ALD, alcohol-associated/related liver disease; ALT, alanine aminotransferase; AST, aspartate aminotransferase; BMI, body mass index; FIB-4, fibrosis-4; GGT, gamma-glutamyl transferase; HbA1c, hemoglobin A1c; HDL, high-density lipoprotein; LDL, low-density lipoprotein; MASLD, metabolic dysfunction-associated steatotic liver disease; MetALD, metabolic dysfunction and alcohol associated steatotic liver disease; SLD, steatotic liver disease.

**Table 2:** Characteristics of non-obese participants divided according to the new SLD nomenclature.

Characteristics	Non-Obese Participants	MASLD	MetALD	Cryptogenic SLD	No SLD	*p*-Value
	(n = 509)	(n = 67)	(n = 4)	(n = 12)	(n = 426)	
Demographics						
Age (years)	45 (35–53)	49 (39–57)	44 (42–46)	41 (28–47)	45 (35–53)	0.056
Male sex	239 (47)	42 (63)	4 (100)	6 (50)	187 (44)	0.005
Alcohol intake, n (%)						<0.001
Mild	198 (39)	33 (49)	0 (0)	4 (33)	161 (38)	
Moderate	36 (7)	0 (0)	4 (100)	1 (8)	31 (7)	
Excessive	5 (1)	0 (0)	0 (0)	0 (0)	5 (1)	
Physical examination						
Waist circumference (cm)	75 (68–80)	81 (77–85)	81 (75–84)	75 (67–80) *	74 (68–78) *	<0.001
BMI (kg/m^2^)	21 (19–23)	24 (22–24)	23 (21–24)	21 (19–22) *	21 (19–23) *	<0.001
Cardiometabolic criteria						
Overweight, n (%)	126 (25)	44 (66)	2 (50)	0 (0)	90 (19)	<0.001
Elevated waist circumference, n (%)	33 (6)	12 (18)	0 (0)	0 (0)	21 (5)	<0.001
Impaired glucose tolerance, n (%)	70 (14)	19 (28)	1 (25)	0 (0)	50 (12)	0.001
Hypertension, n (%)	91 (18)	31 (46)	3 (75)	0 (0)	57 (13)	<0.001
Hypertriglyceridemia, n (%)	114 (22)	38 (57)	3 (75)	0 (0)	73 (17)	<0.001
Low HDL cholesterol, n (%)	56 (11)	22 (33)	0 (0)	0 (0)	34 (8)	<0.001
Laboratory test						
AST (IU/L)	17 (15–21)	19 (17–22)	24 (20–28)	17 (15–21)	17 (15–21) *	0.002
ALT (IU/L)	16 (12–21)	23 (15–30)	39 (22–47)	18 (15–25)	15 (12–21) *	<0.001
GGT (IU/L)	17 (14–26)	26 (17–37)	39 (28–395)	20 (15–37)	17 (13–23) *	<0.001
Creatinine (mg/dL)	0.75 (0.66–0.90)	0.82 (0.70–0.95)	0.85 (0.85–1.05)	0.71 (0.61–0.84)	0.74 (0.65–0.89) *	0.012
Triglycerides (mg/dL)	90 (64–134)	148 (102–229)	308 (116–508)	73 (61–86) *	85 (60–120) *	<0.001
HDL cholesterol (mg/dL)	64 (55–76)	50 (44–60)	46 (43–48)	65 (52–79) *	64 (56–75) *	<0.001
LDL cholesterol (mg/dL)	110 (92–129)	127 (109–134)	148 (115–180)	109 (96–141)	109 (89–124) *	<0.001
HbA1c (%)	5.3 (5.2–5.5)	5.4 (5.3–5.7)	5.3 (5.1–5.3)	5.2 (5.2–5.4) *	5.3 (5.2–5.5) *	<0.001
Platelet (×10^3^/μL)	259 (222–292)	269 (238–310)	258 (186–310)	248 (224–285)	251 (218–287) *	0.013
FIB-4 index	0.74 (0.56–1.01)	0.65 (0.55–0.90)	0.73 (0.58–0.89)	0.64 (0.49–0.88)	0.76 (0.57–1.06)	0.112

Values are presented as numbers (percentages) or medians (interquartile range). * *p* < 0.05, compared to those with MASLD using the Steel–Dwass test for pairwise comparisons. Abbreviations: ALT, alanine aminotransferase; AST, aspartate aminotransferase; BMI, body mass index; FIB-4, fibrosis-4; GGT, gamma-glutamyl transferase; HbA1c, hemoglobin A1c; HDL, high-density lipoprotein; LDL, low-density lipoprotein; MASLD, metabolic dysfunction-associated steatotic liver disease; MetALD, metabolic dysfunction and alcohol associated steatotic liver disease; SLD, steatotic liver disease.

**Table 3:** Characteristics of lean participants divided according to the new SLD nomenclature.

Characteristics	Participants	MASLD	MetALD	Cryptogenic SLD	No SLD	*p*-Value
	(n = 383)	(n = 23)	(n = 2)	(n = 12)	(n = 426)	
Demographics						
Age (years)	43 (34–52)	42 (38–51)	44 (44–44)	41 (28–47)	43 (34–53)	0.683
Male sex	144 (38)	11 (48)	2 (100)	6 (50)	125 (36)	0.142
Alcohol intake, n (%)						<0.001
Mild	128 (33)	10 (43)	0 (0)	4 (33)	114 (33)	
Moderate	28 (7)	0 (0)	2 (100)	1 (8)	25 (7)	
Excessive	5 (1)	0 (0)	0 (0)	0 (0)	5 (1)	
Physical examination						
Waist circumference (cm)	72 (67–76)	77 (76–81)	77 (74–81)	75 (67–80)	71 (66–76) *	<0.001
BMI (kg/m^2^)	20 (19–22)	22 (21–22)	22 (21–23)	21 (19–22)	20 (19–22) *	<0.001
Cardiometabolic criteria						
Overweight, n (%)	0 (0)	0 (0)	0 (0)	0 (0)	0 (0)	NA
Elevated waist circumference, n (%)	19 (5)	4 (17)	0 (0)	0 (0)	15 (4)	0.036
Impaired glucose tolerance, n (%)	41 (11)	6 (26)	0 (0)	0 (0)	35 (10)	0.058
Hypertension, n (%)	55 (14)	11 (48)	2 (100)	0 (0)	42 (12)	<0.001
Hypertriglyceridemia, n (%)	55 (14)	10 (43)	2 (100)	0 (0)	43 (12)	<0.001
Low HDL cholesterol, n (%)	22 (6)	4 (17)	0 (0)	0 (0)	18 (5)	0.078
Laboratory test						
AST (IU/L)	17 (15–21)	18 (16–21)	24 (22–25)	17 (15–21)	17 (15–20)	0.216
ALT (IU/L)	15 (12–19)	18 (13–27)	40 (32–47)	18 (15–25)	15 (12–19)	0.002
GGT (IU/L)	16 (13–21)	20 (13–30)	270 (29–510)	20 (15–37)	16 (13–20)	0.011
Creatinine (mg/dL)	0.73 (0.64–0.85)	0.75 (0.69–0.89)	0.85 (0.85–0.85)	0.71 (0.61–0.84)	0.73 (0.64–0.85)	0.348
Triglycerides (mg/dL)	83 (59–114)	123 (93–201)	308 (198–417)	73 (61–86) *	81 (57–109) *	<0.001
HDL cholesterol (mg/dL)	67 (58–78)	54 (47–62)	45 (42–47)	65 (52–79)	68 (59–79) *	<0.001
LDL cholesterol (mg/dL)	108 (90–1239)	119 (105–131)	179 (178–180)	109 (96–141)	107 (88–123)	0.016
HbA1c (%)	5.3 (5.2–5.5)	5.5 (5.4–5.7)	5.3 (5.3–5.3)	5.2 (5.2–5.4) *	5.3 (5.2–5.5) *	<0.001
Platelet (×10^3^/μL)	249 (218–285)	260 (243–306)	239 (176–302)	248 (224–285)	249 (215–285)	0.348
FIB-4 index	0.74 (0.56–1.04)	0.67 (0.55–0.82)	0.74 (0.57–0.91)	0.64 (0.49–0.88)	0.75 (0.56–1.06)	0.277

Values are presented as numbers (percentages) or medians (interquartile range). * *p* < 0.05, compared to those with MASLD using the Steel–Dwass test for pairwise comparisons. Abbreviations: ALT, alanine amino transferase; AST, aspartate aminotransferase; BMI, body mass index; FIB-4, fibrosis-4; GGT, gamma-glutamyl transferase; HbA1c, hemoglobin A1c; HDL, high-density lipoprotein; LDL, low-density lipoprotein; MASLD, metabolic- dysfunction-associated steatotic liver disease; MetALD, metabolic dysfunction and alcohol associated steatotic liver disease; NA, not available; SLD, steatotic liver disease.

## Data Availability

The datasets generated and/or analyzed during the current study are available from the corresponding author upon reasonable request.
